# Five year outcomes in a cohort study of physicians treated for substance use disorders in the United States

**DOI:** 10.1136/bmj.a2038

**Published:** 2008-11-04

**Authors:** A Thomas McLellan, Gregory S Skipper, Michael Campbell, Robert L DuPont

**Affiliations:** 1 Treatment Research Institute, 600 Public Ledger Building, 150 S Independence Mall, Philadelphia, PA 19106, USA; 2 Alabama Physician Assistance Program, Montgomery, AL, USA; 3 Institute for Behavior and Health, Rockville, MD, USA

## Abstract

**Objective** To evaluate the effectiveness of US state physician health programmes in treating physicians with substance use disorders.

**Design** Five year, longitudinal, cohort study.

**Setting** Purposive sample of 16 state physician health programmes in the United States.

**Participants** 904 physicians consecutively admitted to one of the 16 programmes from September 1995 to September 2001.

**Main outcome measures** Completion of the programme, continued alcohol and drug misuse (regular urine tests), and occupational status at five years.

**Results** 155 of 802 physicians (19.3%) with known outcomes failed the programme, usually early during treatment. Of the 647 (80.7%) who completed treatment and resumed practice under supervision and monitoring, alcohol or drug misuse was detected by urine testing in 126 (19%) over five years; 33 (26%) of these had a repeat positive test result. At five year follow-up, 631 (78.7%) physicians were licensed and working, 87 (10.8%) had their licences revoked, 28 (3.5%) had retired, 30 (3.7%) had died, and 26 (3.2%) had unknown status.

**Conclusion** About three quarters of US physicians with substance use disorders managed in this subset of physician health programmes had favourable outcomes at five years. Such programmes seem to provide an appropriate combination of treatment, support, and sanctions to manage addiction among physicians effectively.

## Introduction

About 10-12% of physicians in the United States develop a substance use disorder.^1^ Those whose condition is detected and who are confronted—typically by their colleagues—are usually referred to the state physician health programmes for intervention, treatment, professional support, and long term monitoring. These programmes began in the United States in the 1970s as volunteer groups of physicians to assist colleagues with alcohol, drug, and mental health problems. The groups evolved into formal agencies operating under the authority of state physician licensing boards. The aim was to reduce public health problems caused by “impaired physicians” through early detection, treatment, and monitoring of substance use and other health problems that cause impairment.

Affected physicians typically are referred to one of these programmes by colleagues or regulatory agencies (for example, hospital board, state licensing board) under the allegation or formal charge of “impaired performance” due to problems with substance use or mental health. Once a formal evaluation has determined that a physician has a problem, a signed contract is arranged under which no further reporting or action occurs pending satisfactory completion of formal treatment, followed by five years of regular monitoring (urine testing).^2^ Thus there is a substantial incentive for physicians to complete treatment and monitoring to enable them to continue practising medicine.^3^

It is important to emphasise that the physician health programmes do not treat physicians. They provide evaluation and diagnosis, develop a contract detailing treatment or monitoring, coordinate and facilitate formal treatment and ongoing professional support, and carry out regular monitoring through random visits to places of work and regular screenings for alcohol and drugs—typically for five years.^2^ The programmes also act as intermediaries between the physician and various regulatory parties (for example, licensing boards, insurers, hospital practice boards). Although variability in the number of affected physicians across the 49 active state programmes (5 to 180 physicians) is considerable, the average programme manages about 65-75 physicians, at an operating annual cost of about $521 000 (£301 000; €381 000)—paid for primarily through a charge appended to all physicians’ licensing fees (about $23 for each physician in most states). These operating costs do not, however, include formal treatment costs for the physicians. Such costs range from about $5000 to $40 000 (depending on the mix of outpatient and residential care) and are typically paid for by a combination of the physician’s health insurance and out of pocket expenses. A full report of the structure and function of physician health programmes is documented in a companion article.^2^

Given the potential public health and safety problems caused by addiction among physicians, evaluation of the effectiveness of care and supervision provided by physician health programmes is important. This is made more important by the fact that studies of these programmes have been short term self evaluations of individual programmes.^4^ ^5^ ^6^ ^7^ Although most of the results from these studies have been positive, few have been independently audited or have provided longer term results.^5^ ^7^ These are important gaps in information. Colleagues need to know whether it is prudent to refer a physician to one of these programmes. Licensing boards, insurers, and patients have the right to know the effectiveness of these programmes. Outcomes from mainstream addiction treatment in the general population have consistently shown poor compliance rates during treatment, and relapse rates of 40-60% within six months of completing treatment.^8^ ^9^ ^10^ ^11^ ^12^

We carried out a two stage independent evaluation of physician health programmes in the United States. In the first study we surveyed 42 of 49 active programmes, describing the personal and professional histories, substance use, and other health problems of the physicians, as well as the intervention, evaluation, referral for treatment, and monitoring activities after treatment provided by those programmes.^2^ This second longitudinal cohort study reports the outcomes in 904 physicians consecutively admitted to a purposive sample of 16 of those programmes and followed for five years. We focused on three outcomes, measured through audited medical records and laboratory tests: completion of the programme, continued alcohol and drug misuse, and occupational status at five years.

## Methods

We carried out a retrospective, longitudinal, cohort study over five years from 1 September 1995 to 1 September 2001 of all physicians consecutively admitted to one of the 16 physician health programmes sampled. We examined the laboratory and medical records of the physicians during the five years.

### Setting, participants, and outcomes

The 42 programmes that participated in the first study were eligible to participate in this second study. Requirements were the availability of records on alcohol and drug testing in a computer analysable format (n=20) and the ability to retrieve information on personal characteristics, participation in treatment, and outcomes of the physicians from their records (n=16). All eligible programmes were included in the study.

The programmes that did and did not participate in the follow-up study were not statistically or clinically significantly different for evaluation, referral, treatment, supervision, support, and monitoring practices. The 16 participating programmes tended to be large: 31% were in the largest quarter of programmes. The mean number of physicians in each programme was 76 (range 11-119).^2^ Although these 16 programmes may not be considered nationally representative, they showed no obvious clinical, administrative, or organisational differences from those not participating.

All 904 physicians with a diagnosis of substance misuse or dependence admitted to the 16 programmes from 1 September 1995 to 1 September 2001 met the inclusion criteria. We restricted the evaluation to objective data from official records (for example, treatment services, attendance, sanctions by the programme, reports to licensing boards) and from laboratory records (urine tests and other specimens).

### Review of medical records

To protect the confidentiality of the physicians, data were collected by members of each programme’s medical records department. Data were collected between November 2006 and January 2007 under training, supervision, and monitoring by the authors.

#### Lost to follow-up

During the study 102 of the 904 (11.3%) participants moved out of their state programme’s jurisdiction. We had no access to any continuing records for those participants (figure[Fig fig1]). We therefore carried out analyses on the remaining 802 physicians.

**Figure fig1:**
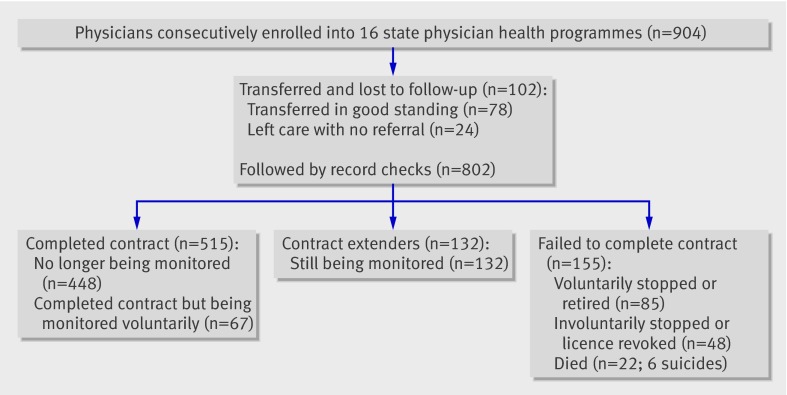
Flow of physicians through trial

#### Missing data

As we used official records there were few instances of missing data (<4%). We report simple descriptive statistics with no substitution procedures for missing values.

### Participants’ characteristics

The participants were predominantly men (87%). The average age was 44 years. Sixty three per cent were married, 18.0% were divorced or separated, and 15.9% were single. Five medical specialties represented more than 50% of physicians: family medicine (20.0%), internal medicine (13.1%), anaesthesiology (10.9%), emergency medicine (7.1%), and psychiatry (6.9%).

The primary drug problems were alcohol (50.3%), opiates (35.9%), stimulants (7.9%), or other substances (5.9%). Fifty per cent reported misusing more than one substance and 13.9% reported a history of intravenous drug use. The average duration of substance misuse was five years. Seventeen per cent had been arrested for an alcohol or drug related offence and 17.0% reported previous treatment for substance misuse.

Fifty five per cent of participants were formally mandated to enter the physician health programme by a licensing board, hospital, insurer, or other agency. The remaining 45% were informally mandated by families, colleagues, employers, or some combination, with the implicit threat of formal action pending results of care.

### Clinical and monitoring procedures

The typical programme consisted of two stages: formal treatment at a specialty addiction programme followed by supervision after treatment.^2^

#### Formal treatment

Overall, 78% of physicians entered residential treatment (mean 72 days, range 30-90), always followed by outpatient treatment (1-3 nights a week) for a recommended 6-9 months. The remaining 22% went directly to outpatient treatment. With good compliance and positive progress, physicians were eligible to return to work under supervision about six months after the start of treatment.

Regardless of setting or duration, most treatment (95%) comprised 12 steps, with the goal of total abstinence from alcohol and other drugs of misuse.^13^ ^14^ The physicians were expected to attend Alcoholics Anonymous or other 12 step groups (92%). Most were encouraged to attend meetings of the Caduceus Society (support group for healthcare providers’ recovery) throughout the remaining years of their contract.

Use of addiction pharmacotherapy as a component of treatment was rare. Only one of these physicians was prescribed methadone for an opiate misuse problem; 46 (6%) were prescribed naltrexone for treatment of alcohol or opiate misuse. In total, 257 physicians (32%) were prescribed an antidepressant for comorbid depression or anxiety disorders.

#### Supervision after treatment

After completion of formal treatment the physicians received coordinated monitoring in several forms.

#### Alcohol and drug testing

A key component of monitoring was random drug testing. Urine was tested in 99.2% of physicians, with rare use of hair (0.2%), saliva (0.1%), and breath (0.6%). Participants were required to phone their programme each workday to find out whether they should report for testing that day, on the basis of random selection. Testing was carried out four times a month early in care, tapering to one or two times a month throughout the monitoring period. The frequency of monitoring was, however, contingent on results of urine testing and compliance with other elements of the care plan.

About 75% of urine sample collections were directly observed by staff responsible for collection. In other cases dry room collection procedures were used. Testing included more than the usual five substance test panel. A typical panel covered 20 substances, including amphetamines, barbiturates, benzodiazepines, opiates, several opioids, cocaine, cannabinoids, and ethyl alcohol.

#### Additional monitoring and response to problems

Participants were expected to attend scheduled appointments for clinical evaluation. They also received random, unannounced visits at their place of work from programme monitors.

If substance use was detected by testing or identified from any other source, almost all the programmes reacted clinically, with combinations of re-evaluation (54%), increased monitoring (43%), and intensified treatment (46%). Forty two per cent of programmes also reported the incident to a licensing board, hospital, or other entity, and an additional 16% started confidential probationary periods without referral to the licensing board or other agency.

The same increases in monitoring occurred for physicians with more than one incident of substance misuse, but almost always with a formal report to the licensing board. In turn, licensing boards did one or more of the following: limited practice (n=129), temporarily suspended the licence (n=94), revoked the licence (n=32), placed the physician’s name in the national monitoring databank (n=121), or restricted the licence to limit prescription of controlled drugs, such as opioids and benzodiazepines (n=56).

## Results

Overall, 102 of 904 physicians (11.3%) moved out of their physician health programme’s jurisdiction and were lost to follow-up. Seventy eight had transferred to another state programme and these physicians were of generally good status at the time of transfer. The remaining 24 moved away without contacting the programme and with no record of referral. This suggests an attempt to avoid monitoring and treatment. As a result of the lack of records for five years on these participants they were lost to follow-up.

Of the remaining 802 physicians with known outcomes, 155 (19.3%) failed to complete their contracted period of formal treatment and supervision. More than half of these (n=85) voluntarily stopped their licences during monitoring owing to factors such as advanced age, financial problems, or psychiatric or other health problems. An additional 48 physicians who failed to complete their contract had their licences revoked owing to significant relapse, usually accompanied by a failure to accept treatment or monitoring. Twenty two physicians died (six from suicide) during participation and another eight died by the five year follow-up (table 1).

**Table 1 tbl1:** Occupational status of physicians at five year follow-up of being in a state physician health programme for substance use disorders. Values are numbers (percentages) of participants

Variable	Completed contract (n=515)	Contract extended (n=132)	Failed to complete contract (n=155)	Followed sample (n=802)
Licensed or practising medicine	477 (92)	97 (73)	15 (10)	589 (73)
Licensed or working (not clinical)	13 (3)	12 (9)	17 (11)	42 (5)
Retired or left practice voluntarily	7 (1)	3 (2)	18 (12)	28 (4)
Licence revoked	9 (2)	14 (11)	64 (41)	87 (11)
Died	3 (1)	0 (0)	27 (17)	30 (4)
Unknown	6 (1)	6 (5)	14 (9)	26 (3)

In total, 515 physicians (64.2%) completed their contracted period. Sixty seven of these physicians voluntarily elected to continue being monitored after completion of their contract, usually as a hedge against relapse.

One hundred and thirty two physicians (16.5%) had their contracts extended beyond the monitoring period. The reasons for continued monitoring included relapse (low severity of relapse that did not endanger patients); failure to comply with requirements, such as group attendance or therapy, or, in some cases, because of previous relapse.

### Alcohol and other drug use during supervision

After formal treatment the physicians were permitted to return to practice under monitoring. Table 2 summarises the results of drug testing for the 647 physicians who completed their contract or had their contracts extended; 81% of the total group. Fifty seven of 515 physicians who completed their contract and 69 of 132 physicians with extended contracts had a positive alcohol or drug test result at some point during a mean of 56 months, with testing twice a month. Across both groups 33 of the 126 with a positive test result retested positive.

**Table 2 tbl2:** Results of drug tests throughout monitoring period for 647 physicians who completed their contract with a physician health programme or had it extended

Variable	Completed contract (n=515)	Contract extended (n=132)	Both groups (n=647)
Average duration of contract (months)	54	64	56
Mean No of drug tests per physician	82	121	94
No (%) with at least one positive drug test result	57 (11)	69 (52)	126 (19)
No (%) with a repeat positive result*	8 (16)	25 (38)	33 (26)

*Percentage of those with one positive test result.

Of 159 documented incidents of substance misuse (126 initial positive test results and 33 repeat positive results, across both groups), 10 (6%) were in the context of medical practice, such as on duty or on call. One episode of patient harm (over-prescribing drugs) was noted in the records. These records, however, captured only important, detected negative consequences. It was not possible to capture other important but less obvious consequences of substance use not recorded in the charts.

### Occupational status after supervision

Records showed that five years after the start of the contracts 631 of the 802 physicians (78.7%) were licensed without restriction and either practising medicine or working in a non-clinical capacity (table 1). An additional 28 physicians (4%) had retired or voluntarily left practice, 87 (11%) had their licence revoked, 30 (4%) had died (7 substance misuse, 6 suicides, 17 other), and 26 (3%) had missing data.

The status of the physicians at five year follow-up varied as a function of their completion status of the physician health programme. For example, 95% of physicians who had completed their contract and 82% who had their contract extended were still licensed and less than 1% had died. In contrast, 21% of the physicians who did not complete their contract were still licensed and 17% had died.

## Discussion

About three quarters of US physicians treated for substance use disorders in physician health programmes had favourable outcomes throughout five years. Such programmes seem to provide an appropriate combination of treatment, support, and sanctions to manage addiction among physicians effectively.

Physician health programmes share the dual role of helping addicted physicians attain sobriety and personal recovery as well as providing assurance to colleagues, hospitals, insurers, licensing boards, and the general public that these physicians can practise safe care. The processes used by these programmes include clinical assessment, referral for treatment, and support and monitoring after treatment, usually for five years. Many questions have been raised about the effectiveness of these programmes—one was stopped owing to allegations of poor monitoring.^15^ ^16^

We carried out a longitudinal, retrospective cohort study of 904 physicians consecutively admitted to 16 state physician health programmes. Objective outcomes were derived exclusively from laboratory results of urine testing and audit of official records.

All the participants entered some period of professional, specialty treatment, typically 60-90 days in a residential setting, followed by continuing outpatient care. Formal treatment was followed by a return to work conditional on continued participation in 12 step support groups, formal meetings with the programme monitor, random alcohol and drug testing, and random visits by programme staff at the workplace. We know of no comparably intensive or protracted form of treatment and monitoring provided to any other group of addicted people in the United States.^8^ ^9^ ^10^ ^12^

At five year follow-up 14% of the physicians had stopped practising medicine (voluntarily or forcibly) as a result of their identification by and participation in a physician health programme. It is difficult to determine whether this rate for termination of licences is evidence of close monitoring and tough sanctions or inadequate monitoring and lax standards. The urine test results in the 647 physicians who completed their contract with the programme and those whose contract was extended may provide the best evidence. 

Over the average course of 56 months of random testing (about 94 urine tests of 20 panels each), combined with unannounced visits to the physicians’ workplace by a programme monitor, the records showed that 81% had no identified substance misuse at any time. Nineteen per cent, however, had at least one incident of substance misuse during the five years of monitoring. Ten of these incidents were in the context of patient care (on duty or on call) and one instance of patient harm was recorded (over-prescribing drugs). It might be expected that any detection of alcohol or other misuse of drugs would result in immediate suspension or revocation of a licence. This was the case only when there had been a period of non-compliance or if the circumstances of the relapse were dangerous. Instead, most of the programmes in this sample increased the intensity of clinical care as well as the frequency of drug testing and supervisory visits—typically with reports to the licensing board. The more serious sanctions included restrictions on, or suspensions of, the licence or prescription privileges. Evidence suggests that this may be a sensible approach as only 26% of the 126 physicians who tested positive retested positive.

### Limitations

Our study has several limitations. Firstly, the sample cannot be considered nationally representative of physician health programmes in the United States. Because of financial and time constraints we needed to audit primary, objective outcome measures rapidly and efficiently, and only 16 of the 42 programmes that volunteered had electronic clinical and laboratory records continuously available from 2001 to 2007; we selected all of these programmes. That these 16 programmes started to keep electronic records seven years ago suggests that they may have been among the best funded or best led programmes at that time. Data from the phase one survey indicated that the programmes included larger samples (mean census 76 *v* 68), with correspondingly larger budgets; but, importantly, the duration of the programmes, their clinical, administrative, and sanctioning approaches, and the procedural elements of care did not differ.

A second limitation is that we recorded only objective, verifiable information from records, such as drug testing, sanctions, and modifications to licences. We are confident of the validity of these records, and our results are consistent with most other published studies of physicians with substance use disorders.^4^ ^5^ ^6^ ^7^ However, the official records provide only a limited picture of the broader functional status and personal health of these physicians. A prospective study is needed to enrich these data, with additional information on clinical and administrative processes and a broader range of measures for functional status.

Finally, the focus on official records made it impossible to track 102 physicians who moved out of their programmes’ jurisdiction during the course of care. Although most of these physicians (n=78) were in good standing at the time of transfer, longer term results cannot be inferred. It is a concern that 24 of these physicians moved away without contacting their programme and with no formal referral for continued monitoring. This suggests an effort to avoid detection and is thus a potential danger to patients.

### Conclusions

From a clinical perspective we interpret these results as evidence that the combination of identification, intervention, formal treatment, professional support, and monitoring by physician health programmes is effective in rehabilitating most of these addicted physicians, over at least five years. From a public safety perspective we believe these data indicate that most physicians who could not or would not stop their misuse of substances were detected early during the course of formal treatment and this usually resulted in voluntary or involuntary cessation of practice. From a policy perspective we conclude that affected physicians are well advised to enter the supervision of a physician health programme voluntarily, and that regulatory boards are well advised to continue supporting these programmes.

It is not possible from the evidence here to prove whether this form of support and monitoring for physicians with substance use disorders is appropriate, too harsh, or too permissive. Any episode of substance use in the context of patient care has the potential for considerable harm. Thus it will always feel more powerful to invoke sanctions alone in a “get tough” policy. But sanctions without the prospect of help in achieving recovery could simply reduce colleagues’ willingness to refer affected physicians—or licensing boards to exercise harsh sanctions—potentially increasing the true prevalence of the problem. On the basis of these data, and considering available alternatives, physician health programmes seem to provide the best available measures for protecting patients and for recovering physicians’ careers.

**What is already known on this topic**

10-12% of physicians in the United States become addicted to alcohol and other drugs
Addicted physicians receive treatment through physician health programmes, operating under jurisdiction of state licensing boards

**What this study adds**

Most US physicians with substance use disorders managed in physician health programmes had favourable outcomes at five years
During monitoring 81% had negative urine test results
Most (95%) who completed monitoring were licensed and working as physicians at five years

## Notes

We thank the Robert Wood Johnson Foundation for unrestricted career award funds and the Federation of State Physician Health Programs for its cooperation and access to the data.

Contributors: ATMcL oversaw data collection and audits, participated in the analyses and writing of the paper, and is guarantor. ATMcL and RLDuP designed the study and secured funding. GSS designed the study and data collection, supervised data collection, and participated in the analyses and writing of the paper. GSS and RLDuP liaised and communicated with the Federation of State Physician Health Programs. MC was responsible for the preparation and storage of the data and analyses and participated in the writing of the paper. RLDuP assisted in the design of the data collection instrument, oversaw audits of data, and participated in the analyses and writing of the paper.

Ethical approval: This study was approved by the institutional review board of the Treatment Research Institute, Philadelphia.

## Notes

**Cite this as: ***BMJ* 2008;337:a2038
